# Peak expiratory flow is a reliably household pulmonary function parameter correlates with disease severity and survival of patients with amyotrophic lateral sclerosis

**DOI:** 10.1186/s12883-022-02635-z

**Published:** 2022-03-19

**Authors:** Qi-Jie Zhang, Jian-Chai Huang, Jia Chen, Wei Hu, Liu-Qing Xu, Qi-Fu Guo

**Affiliations:** 1 Department of Neurology, Fujian Institute of Neurology, The First Affiliated Hospital, Fujian Medical University, 20 Chazhong Road, Fuzhou, 350005 China; 2 Department of Respiratory and Critical Care Medicine, The First Affiliated Hospital, Fujian Medical University, Fuzhou, China

**Keywords:** Amyotrophic lateral sclerosis, Peak expiratory flow, Prognosis, Survival

## Abstract

**Background:**

Amyotrophic lateral sclerosis (ALS) is an incurable and fatal neurodegenerative disease; most ALS patients die within 3 to 5 years after symptom onset, usually as a consequence of respiratory failure. In the present study, we aim to screen the survival-related pulmonary function parameters, and to explore the predictive value of peak expiratory flow (PEF) in disease severity and prognosis in patients with ALS.

**Methods:**

The discovery cohort included 202 ALS patients, and the demographic and clinical characteristics of eligible patients were collected and pulmonary function tests were performed using MS-PFT spirometer. In the validation cohort, 62 newly diagnosed ALS patients performed the pulmonary function test by MS-PFT spirometer and household peak flow meter (KOKA) simultaneously.

**Results:**

Among 12 pulmonary function parameters, FVC, FEV1, PEF, MEF75%, and MVV were identified to be independent predictive factors for survival. PEF was highly correlated with FVC (*r* = 0.797), MVV (*r* = 0.877), FEV1 (*r* = 0.847), and MEF75% (*r* = 0.963). Besides, the values of PEF were positively associated with disease severity (ALSFRS-R score, r_s_ = 0.539, *P* < 0.0001), and negatively associated with progression rate (ΔALSFRS-R, r_s_ = -0.316, *P* < 0.0001). Finally, we also confirmed that the values of KOKA-measured PEF were highly correlated with the ones measured using MS-PFT spirometer (*r* = 0.9644, *p* < 0.0001).

**Conclusions:**

Our work emphasizes the critical role of PFTs in predicting prognosis of ALS patients. PEF is an easily available pulmonary function index, which is also a promising indicator in predicting disease severity and survival for ALS patients.

**Supplementary Information:**

The online version contains supplementary material available at 10.1186/s12883-022-02635-z.

## Background

Amyotrophic lateral sclerosis (ALS) is a chronic, incurable and fatal neurodegenerative disease, pathologically characterized by progressive loss of motor neurons in the motor cortex, brainstem, and spinal cord. Clinically, it is featured by adult onset, male predominance, upper limb muscle weakness and atrophy, and relentless progression involving trunk and bulbar muscles [1]. Due to the rapidly progressive loss of motor function, most ALS patients die within 2 to 5 years after symptom onset, usually as a consequence of respiratory failure [2]. The assessment of respiratory function and respiratory care are therefore of great importance for ALS patients [3, 4]. Pulmonary function tests (PFTs) have been frequently applied for evaluating respiratory function [5]. Forced vital capacity (FVC) is the most important respiratory index highly correlated with survival in ALS [6]. For the time being, the pulmonary function tests are mainly performed by respiratory physiotherapists in hospitals with specialized instrumentation (MS-PFT spirometer), and largely depend on the cooperation of ALS patient [7]. For those patients at the later stage of ALS, the pulmonary function tests are hardly carried out due to their unwillingness or inability to come to the hospital owing to their mobility inconvenience. So it is necessary to explore more practical and feasible pulmonary function indexes for the prediction of progression and survival in ALS. Peak expiratory flow (PEF) is another useful pulmonary function index, which was defined as the maximum expiratory flow per minute. Advantageously, PEF can be measured by a household peak flow meter, which is simple in structure, easy to use, cheap, and can be self-monitored at home [8, 9]. In the present study, we aimed to screen the survival-related pulmonary function parameters, and subsequently explored the predictive value of PEF in disease severity and prognosis for ALS.

## Methods

### Subjects

For the discovery cohort, 221 ALS patients were recruited serially from the Department of Neurology, First Affiliated Hospital of Fujian Medical University from Dec. 2014 to Sep. 2019. For the validation cohort, 62 newly diagnosed ALS patients were enrolled from Oct. 2020 to Apr. 2021. All the patients fulfilled the diagnostic criteria of ALS according to the revised Escorial criteria, and the diagnosis was confirmed by two professional neurologists [10]. Written informed consent was obtained from each participant. Patients who did not complete pulmonary function examination and refused to participate were excluded. This study was approved by the Ethics Board of the First Affiliated Hospital of Fujian Medical University and performed in accordance with the declaration of Helsinki.

### Clinical data collection

Clinical profiles were recorded at the time of diagnosis, including demographic characteristics, age of onset, site of onset, body mass index, smoking history, history of hypertension and diabetes mellitus, family history of ALS, diagnostic category, diagnostic delay, and treatments. Smoking history was defined as smoking at least one cigarette per day, for more than 6 months. Diagnostic delay was defined as the time interval from disease onset to identified diagnosis of ALS. The revised ALS functional rating scale (ALSFRS-R) was used to assess the severity of the disease at the time of pulmonary function test. The linear change rate of ALSFRS-R (ΔALSFRS-R) was used to reflect disease progression, and calculated by the equation: ΔALSFRS-R = (48—ALSFRS-R score) / duration from disease onset to pulmonary function test in months. The use of riluzole was defined as treatment with riluzole (50 mg, twice per day) for longer than 3 months. The use of noninvasive positive pressure ventilation (NIPPV) was defined as an acceptance of NIPPV support by patients for longer than 4 h per day. For survival analysis, all the enrolled cases were followed up at least more than 24 months. The primary endpoint was defined by either death or tracheotomy.

### Pulmonary function tests

All pulmonary function tests were performed with an MS-PFT spirometer (Jaeger, Germany), and carried out by the same experienced respiratory physiotherapist in compliance with the American Thoracic Society/European Respiratory Society guidelines [11, 12]. A total of 12 pulmonary function parameters (expressed as the percentage of predicted values (%), except forced expiratory volume in 1 s (FEV1)/FVC presented as the measured value) were analyzed: tidal volume (VT), expiratory reserve volume (ERV), FVC, FEV1, FEV1/FVC, PEF, maximal expiratory flow at 75% of FVC (MEF75%), maximal expiratory flow at 50% of FVC (MEF50%), maximal expiratory flow at 25% of FVC (MEF25%), maximal mid-expiratory flow (MMEF), minute ventilation (MV) and maximal voluntary ventilation (MVV). In the validation cohort, 62 newly diagnosed ALS patients accepted the pulmonary function test by MS-PFT spirometer and household peak flow meter (KOKA) simultaneously. During pulmonary function test, the patient was encouraged to optimize performance. At least three acceptable and repeatable trials were performed, and the best out of these manoeuvres was chosen.

### Statistical analysis

A statistical analysis was performed using the SPSS statistical software version 20.0 for Windows (SPSS, Inc, IL, USA). All continuous variables were examined for normal distribution and homogeneity test of variance prior to analysis, and presented as mean ± standard deviation. All categorical variables were expressed as number (percentage) and analyzed using chi-square test. The pulmonary function parameters were described by tertiles, selected by log rank test of Kaplan Meier analysis, and then adjusted using Multivariate Cox regression analysis. X-tile software was adopted to get the cut-off value. Correlations were calculated with the Pearson coefficient (r) or Spearman coefficient (r_s_). *P* value less than 0.05 was considered statistically significant.

## Results

### Clinical characteristic of ALS patients

A total of 221 ALS patients with pulmonary function testing were serially enrolled in the department of neurology. Among them, 3 cases were combined with malignant tumors, including 1 patient with nasopharyngeal carcinoma, 1 patient with glioma, and 1 patient with small cell lung cancer. Additionally, 2 cases were combined with chronic obstructive pulmonary disease, and 1 case with chronic bronchiectasis. Three patients could not cooperate well during pulmonary function testing. Ten patients were lost during follow up.

The remaining 202 ALS patients were included into the final analysis. Among them, 180 cases performed the pulmonary function test at the time of disease diagnosis, and the remaining 22 cases underwent the first pulmonary function assessment during following-up. There were 129 males and 73 females (ratio of male to female was 1.77:1). The mean age of disease onset was 54.28 ± 11.00 years old. The median diagnostic delay was 11.0 months. According to the site of first symptom onset, 43 cases were bulbar onset, and the remaining 159 cases were spinal onset. The descriptive analysis results of demographic and clinical features are shown in Table 1.

**Table 1:** Clinical Characteristics of patients with ALS

variables and subgroups	No.(%),mean ± SD or median (IQR)
Gender	
male	129(63.86%)
female	73(36.14%)
Family history	
yes	10(4.95%)
no	192(95.05%)
Age of onset, mean ± SD, y	54.28 ± 11.00
Site of onset	
bulbar	43(21.29%)
upper limb	111(54.95%)
lower limb	48(23.76%)
Diagnostic delay, median (IQR), mo	11.0(6.0,17.0)
BMI at baseline, mean ± SD, kg/m2	21.71 ± 2.78
ALSFRS-R score, median (IQR)	40(36,44)
ΔALSFRS-R, median (IQR)	0.55(0.32,1.13)
Use of riluzole	
yes	156(77.23%)
no	46(22.77%)
PEG	
yes	19(9.41%)
no	183(90.59%)
NIPPV	
yes	44(21.78%)
no	158(78.22%)
Smoking	
yes	84(41.58)
no	118(58.42%)
Hypertension	
yes	26(12.87)
no	176(87.13)
Diabetes mellitus	
yes	20(9.90%)
no	182(90.10%)

*Abbreviation: ALS* Amyotrophic Lateral Sclerosis, *BMI* Body Mass Index, *ALSFR-R* Amyotrophic Lateral Sclerosis Functional Rating Scale–Revised, *PEG* Percutaneous Endoscopic Gastrostomy, *NIPPV* Noninvasive Positive Pressure Ventilation, *IQR* Interquartile Range, *SD* Standard Deviation

For survival analysis, 149 (73.76%) patients reached the endpoint of this study, of which 136 cases died and 13 cases underwent tracheotomy. The remaining 53 (26.24%) patients were still alive at the last follow-up visit. The mean survival time was 39.69 ± 23.84 months (median survival time was 37 months). The survival analysis showed that an older age of onset (≥ 55 y), shorter diagnostic delay (< 9 m), lower BMI at baseline (< 19.20 kg/m^2^), and faster progression rate (ΔALSFRS-R ≥ 0.56) were highly linked with shorter survival (supplemental Fig. 1A-D), which was consistent with previous studies [13–15]. No significant differences were found between survival time and gender (male vs. female), use of riluzole (yes vs. no), site of onset (bulbar vs. upper limb vs. lower limb), and ALSFRS-R score at baseline (supplemental Fig. 1E–H), which was similar to the results we have been reported before [16].

**Fig. 1:**
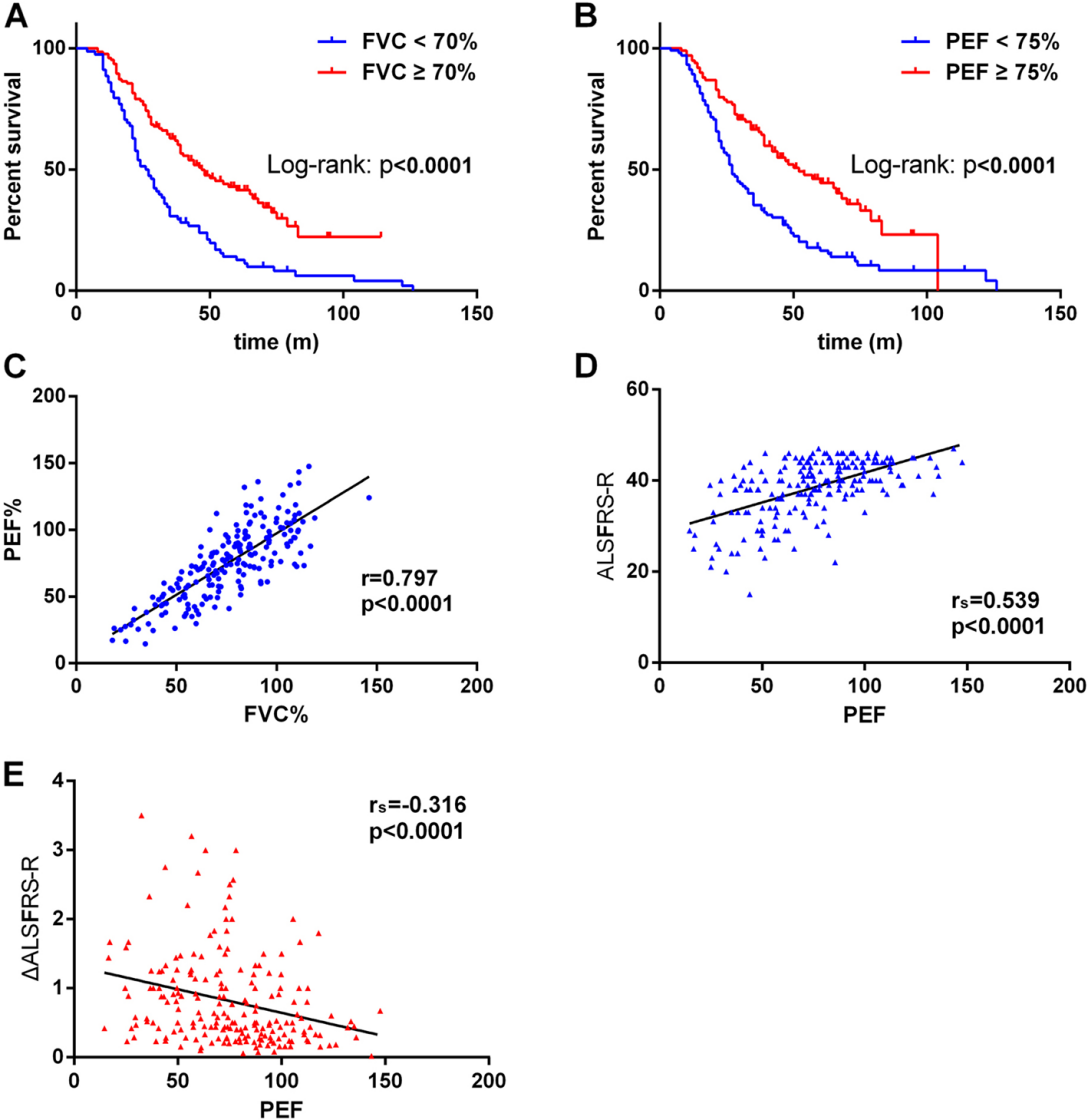
**A** The Kaplan–Meier survival curves of ALS patients with FVC < 70% vs. FVC ≥ 70%. **B** The Kaplan–Meier survival curves of ALS patients with PEF < 75% vs. PEF ≥ 75%. **C** Correlation analysis of PEF and FVC. **D** The values of PEF were positively correlated with ALSFRS-R score. **E** The values of PEF were negatively associated with ΔALSFRS-R

### ALS survival-related pulmonary function parameters

Firstly, Log rank test of Kaplan Meier analysis was adopted to screen the significant pulmonary function indexes that affect the survival of ALS patients. We found that 7 parameters were statistically significant, including FVC, FEV1, FEV1/FVC, PEF, MEF75%, MEF50%, and MVV (*p* < 0.05, supplemental Table 1). Subsequent multivariate Cox regression analysis was used to identify the survival-related pulmonary function parameters after adjustment for sex, age of onset, site of onset, body mass index (BMI), ALSFRS-R score at pulmonary function test, progression rate (ΔALSFRS-R), time interval between disease onset and pulmonary function test, use of riluzole, acceptance of PEG, use of NIPPV, as well as the history of smoking, hypertension, and diabetes mellitus. This analysis indicated that FVC, FEV1, PEF, MEF75%, and MVV were independent predictive factors for survival in ALS patients (Table 2). The cut-off values of FVC, FEV1, PEF, MEF75%, and MVV were 70%, 75%, 75%, 90%, and 55% respectively, which were identified using X-tile software (Fig. 1A-B, supplemental Fig. 2A-C).

**Table 2:** The pulmonary function parameters that significantly associated with survival after adjustment for possible influencing factors^a^

pulmonary function parameter	β	SE	Wald	P*	*HR*(95%CI)*
FVC					
> 86.42					1.000
66.82–86.42	0.467	0.232	4.068	0.044	1.595(1.013,2.511)
< 66.82	1.202	0.245	24.045	< 0.001	3.326(2.057,5.377)
FEV1					
> 90.74					1.000
72.62–90.74	0.277	0.223	1.537	0.215	1.319(0.851,2.044)
< 72.62	1.286	0.239	28.909	< 0.001	3.618(2.264,5.781)
PEF					
> 87.40					1.000
63.18–87.40	0.679	0.230	8.696	0.003	1.971(1.256,3.094)
< 63.18	1.339	0.259	26.828	< 0.001	3.816(2.299,6.334)
MEF75%					
> 91.02					1.000
66.26–91.02	0.639	0.222	8.270	0.004	1.894(1.226,2.928)
< 66.26	0.824	0.251	10.765	0.001	2.280(1.393,3.730)
MVV					
> 71.15					1.000
42.41–71.15	0.925	0.230	16.164	< 0.001	2.522(1.607,3.959)
< 42.41	1.460	0.251	33.780	< 0.001	4.308(2.632,7.049)

*Abbreviation: HR* Hazard Ratio, *FVC* Forced Vital Capacity, *FEV1* Forced Expiratory Volume in 1 s, *PEF* Peak Expiratory Flow, *MEF75%* Maximal Expiratory Flow at 75% of FVC, *MVV* Maximal Voluntary Ventilation

^a^ Possible influencing factors included sex, age of onset, site of onset, BMI, use of riluzole, acceptance of PEG, use of NIPPV, smoking, hypertension, diabetes mellitus, ALSFRS-R score at pulmonary function test, progression rate (ΔALSFRS-R), and time interval between disease onset and pulmonary function test

^*^ Multivariate Cox survival analysis, Backward Stepwise (Wald)

**Fig. 2:**
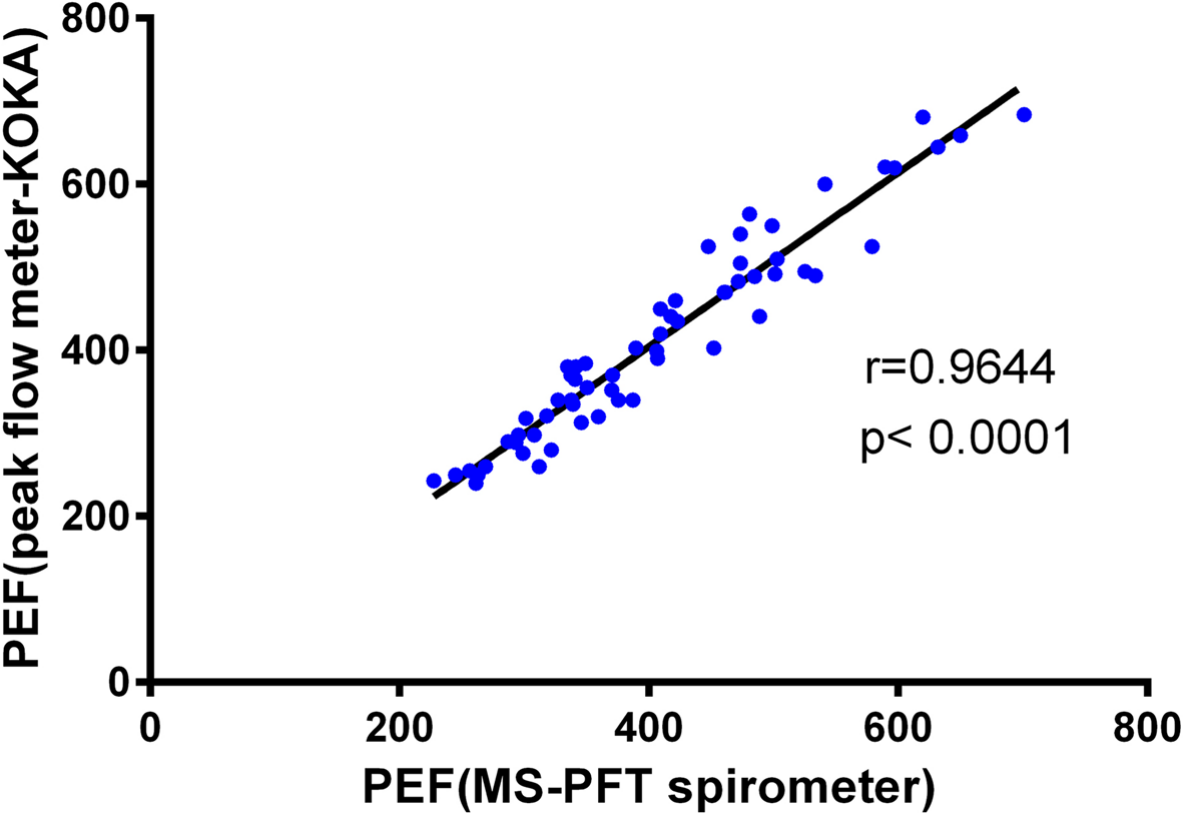
Correlation analysis of PEF measured by household peak flow meter (KOKA) and MS-PFT spirometer

### PEF predicts disease severity and prognosis

In this cohort of ALS patients, except for FVC, we observed four additional survival-relevant pulmonary function parameters, including PEF, FEV1, MEF75%, and MVV. PEF was also highly linked to FVC (*r* = 0.797, *p* < 0.0001, Fig. 1C), FEV1 (*r* = 0.847, *p* < 0.0001, supplemental Fig. 2D), MEF75% (*r* = 0.963, *p* < 0.0001, supplemental Fig. 2E), and MVV (*r* = 0.877, *p* < 0.0001, supplemental Fig. 2F). Furthermore, the values of PEF were positively associated with disease severity (ALSFRS-R score, r_s_ = 0.539, *P* < 0.0001), and negatively associated with progression rate (ΔALSFRS-R, r_s_ = -0.316, *P* < 0.0001) (Fig. 1E–F).

### Effective measurement of PEF using household peak flow meter

To explore the efficiency of PEF measured by household peak flow meter, we also designed a validation cohort including 62 newly diagnosed patients with ALS, who accepted the pulmonary function test by MS-PFT spirometer and household peak flow meter (KOKA) simultaneously. The values of KOKA-measured PEF were highly correlated with the ones measured using MS-PFT spirometer (*r* = 0.9644, *p* < 0.0001, Fig. 2).

## Discussion

Most ALS patients tend to develop progressive respiratory muscle weakness and paralysis, leading to respiratory failure, which is the principal cause of death. At the early stage, patients often report nocturnal hypoventilation symptoms, such as morning headache, daytime sleepiness and loss of concentration or memory. However, those symptoms are not specific and are easily ignored. Therefore, pulmonary function testing is a powerful avenue for monitoring respiratory status, and FVC is one of the most common measured pulmonary function parameters reflecting lung capacity, which is highly related to disease progression and survival in ALS, and helps the neurologists to determine when to initiate noninvasive ventilation for ALS patients [17, 18]. In the present study, a total of 12 pulmonary function parameters were analyzed in a cohort of 202 ALS patients. After Kaplan Meier and Multivariate Cox regression analysis, FVC, FEV1, PEF, MEF75%, and MVV were identified to be highly associated with survival.

Aiming to explore more practical and feasible pulmonary function index for the prediction of progression and survival in ALS, we put a heavy emphasis on PEF. PEF is another commonly used index to reflect the pulmonary ventilation function, which has received increasing attention as useful measurement in patients with respiratory diseases [9, 19]. PEF can also be used to reflect the expiratory muscle function reliably in patients with neuromuscular disease [20]. The prominent advantage of PEF lies in the feasibility of self-monitoring at home, which could be achieved by a simple household peak flow meter [21, 22]. The advent of smart spirometer has allowed for several pulmonary function parameters (for instance slow vital capacities (SVC), FVC and FEV1); however, compared to the peak flow meter, the cost of smart spirometer examination is relatively higher, and the operation is more complex (necessary to be calibrated with calibration pump), which could limit its wide application to some extent, especially at home. Until now, there is no recommendation with respect to the application of PEF in ALS patients. In order to make home usage-PEF better reflect respiratory function of ALS patients, we had taken the following corresponding measures. Firstly, sufficient education and training were provided during their first visits in hospital, aiming to master the key points of operation in PEF. Secondly, household PEF measure needs the supervision and help of caregiver. Thirdly, it is necessary to repeat the PEF measurement three times at least, and it is also necessary to perform at different time periods (including morning, afternoon and evening), and record the best result. Lastly, for the patients with bulbar involvement, it’s often hard to get the FVC or PEF result correctly, partly due to the air leak during pulmonary function testing. Therefore, we proposed a revised peak flow meter with oronasal mask (supplemental Fig. 3). It is also well known that for those ALS patients at the later stage with bulbar involvement, the noninvasive ventilation support is necessary, and the measurement of PEF is of little significance.

In the present study, we observed that PEF was an independent predictive factor for ALS survival, even after multiple adjustments of sex, age of onset, site of onset, BMI, use of riluzole, ALSFRS-R score at pulmonary function test, progression rate (ΔALSFRS-R), and time interval between disease onset and pulmonary function test. Furthermore, PEF was highly correlated with other ALS survival-related pulmonary function parameters, including FVC, MVV, FEV1, and MEF75%, which showed that PEF was as good as FVC in reflecting lung function in ALS patients. Additionally, the values of PEF were moderately correlated with disease severity (assessed by ALSFRS-R score at baseline) and disease progression rate (expressed as ΔALSFRS-R), which was consistent with the results from Pirola A and colleagues [17]. However, Carvalho et al. recently observed that PEF did not significantly reflect the progression rate of ALS in a small (*n* = 51) but longitudinal ALS cohort [23], and further large and prospective study is necessary to confirm the correlation between PEF and disease progression. Yamada et al. also reported that PEF could be a specific respiratory marker for the severity and progression of patients with spinal and bulbar muscular atrophy, a rare neuromuscular disease, also known as Kennedy’s disease [24]. Besides, the values of PEF could be measured by household peak flow meter (KOKA) effectively. Taken together, the results from this study suggested that PEF may be a second candidate pulmonary function index, which could reliably predict disease progression and survival in ALS patients.

In the pulmonary function test, there are different PFTs that could reflect lung volume, lung ventilation, and small airway functions in clinical. Understanding whether these parameters are related to disease progression and survival in ALS patients is useful for clinical decision. Similar to FVC, SVC has also received recent attention. Paillisse et al. evaluated the pulmonary function parameters in a cohort of 2069 patients, and concluded that SVC was a predictor of survival in ALS [25]. Further studies confirmed that SVC and FVC were very strongly associated and declined similarly in ALS, and were inter-changeable in predicting survival in ALS [26, 27]. Andrews JA et al. also identified that the rate of decline in SVC was correlated with respiratory failure, tracheostomy, or death in patients with ALS [28]. Besides, Pirola A et al. found that supine position FVC (sFVC) and monthly decline of sFVC% were significantly associated with the survival of ALS patients [17]. In our study, we also observed that VC max, which was equal to the maximum value of SVC and FVC, was an important indicator of clinical progression.

Some limitations were also noted in our study. Firstly, some pulmonary function parameters were not mentioned in our study, such as supine position FVC, slow vital capacities, and peak expiratory cough flow. Besides, as a single research center, the sample size was relatively small, and it was not a population-based study, which lead to the presence of admission rate bias. Lastly, the present study was a cross-sectional design, and it is necessary to confirm the application of PEF in future multi-center prospective and longitudinal studies.

## Conclusions

Our work emphasizes the critical role of pulmonary function parameters in predicting prognosis of ALS. PEF could be a practical and household pulmonary function parameter, which is also a promising indicator in predicting disease severity and survival for ALS patients.

## Supplementary Information

**Additional file 1.**

**Additional file 2.** 

## Data Availability

The datasets analyzed during the current study are available in the supplemental materials.

## Abbreviations

ALS: Amyotrophic lateral sclerosis
FVC: Forced vital capacity
PEF: Peak expiratory flow
ALSFRS-R: The revised ALS functional rating scale
NIPPV: Noninvasive positive pressure ventilation
VT: Tidal volume
ERV: Expiratory reserve volume
MEF75%: Maximal expiratory flow at 75% of FVC
MEF50%: Maximal expiratory flow at 50% of FVC
MEF25%: Maximal expiratory flow at 25% of FVC
MMEF: Maximal mid-expiratory flow
MV: Minute ventilation
MVV: Maximal voluntary ventilation
BMI: Body mass index
