# Supplementary material for: Peak expiratory flow is a reliably household pulmonary function parameter correlates with disease severity and survival of patients with amyotrophic lateral sclerosis
Source: BMC Neurol. 2022 Mar 19;22:105. doi: 10.1186/s12883-022-02635-z (PMC8933978; doi:10.1186/s12883-022-02635-z)
Supplement: Supplementary file 1 — Additional file 1. [file 12883_2022_2635_MOESM1_ESM.docx]

**Title:** Peak expiratory flow is a reliably household pulmonary function parameter correlates with disease severity and survival of patients with amyotrophic lateral sclerosis

Table S1 Screening of the pulmonary function indexes that affect the survival of ALS

| Pulmonary function parameter | Groups | Outcome | | Log-rank test | |
| --- | --- | --- | --- | --- | --- |
|  |  | alive | death/ tracheotomy | $\chi^{2}$ | *P* |
| VT |  |  |  | 2.694 | 0.260 |
|  | >214.58 | 22(33.85) | 43(66.15) |  |  |
|  | 154.27-214.58 | 18(27.69) | 47(72.31) |  |  |
|  | <154.27 | 13(20.31) | 51(79.69) |  |  |
| MV |  |  |  | 0.212 | 0.900 |
|  | >256.97 | 21(31.34) | 46(68.66) |  |  |
|  | 171.26-256.97 | 15(22.06) | 53(77.94) |  |  |
|  | <171.26 | 17(25.37) | 50(74.63) |  |  |
| ERV |  |  |  | 1.115 | 0.573 |
|  | >77.98 | 17(30.91) | 38(69.09) |  |  |
|  | 44.79-77.98 | 14(25.45) | 41(74.55) |  |  |
|  | <44.79 | 18(32.73) | 37(67.27) |  |  |
| FVC |  |  |  | 22.429 | <0.001 |
|  | >86.42 | 30(44.78) | 37(55.22) |  |  |
|  | 66.82-86.42 | 20(29.41) | 48(70.59) |  |  |
|  | <66.82 | 3(4.48) | 64(95.52) |  |  |
| FEV1 |  |  |  | 21.904 | <0.001 |
|  | >90.74 | 28(41.79) | 39(58.21) |  |  |
|  | 72.62-90.74 | 22(32.35) | 46(67.65) |  |  |
|  | <72.62 | 3(4.48) | 64(95.52) |  |  |
| FEV1/FVC |  |  |  | 8.951 | 0.011 |
|  | >90.79 | 7(10.45) | 60(89.55) |  |  |
|  | 82.94-90.79 | 25(36.76) | 43(63.24) |  |  |
|  | <82.94 | 21(31.34) | 46(68.66) |  |  |
| PEF |  |  |  | 18.370 | <0.001 |
|  | >87.40 | 32(48.48) | 34(51.52) |  |  |
|  | 63.18-87.40 | 14(20.59) | 54(79.41) |  |  |
|  | <63.18 | 7(10.45) | 60(89.55) |  |  |
| MEF75% |  |  |  | 14.520 | 0.001 |
|  | >91.02 | 31(46.27) | 36(53.73) |  |  |
|  | 66.26-91.02 | 13(19.12) | 55(80.88) |  |  |
|  | <66.26 | 9(13.43) | 58(86.57) |  |  |
| MEF50% |  |  |  | 7.744 | 0.021 |
|  | >87.30 | 26(38.81) | 41(61.19) |  |  |
|  | 64.28-87.30 | 15(22.06) | 53(77.94) |  |  |
|  | <64.28 | 12(17.91) | 55(82.09) |  |  |
| MEF25% |  |  |  | 1.251 | 0.535 |
|  | >88.94 | 20(30.30) | 46(69.70) |  |  |
|  | 61.32-88.94 | 17(25.37) | 50(74.63) |  |  |
|  | <61.32 | 16(24.24) | 50(75.76) |  |  |
| MMFF |  |  |  | 5.448 | 0.066 |
|  | >82.49 | 25(37.31) | 42(62.69) |  |  |
|  | 62.59-82.49 | 15(22.39) | 52(77.61) |  |  |
|  | <62.59 | 13(19.70) | 53(80.30) |  |  |
| MVV |  |  |  | 20.419 | <0.001 |
|  | >71.15 | 33(49.25) | 34(50.75) |  |  |
|  | 42.41-71.15 | 16(23.88) | 51(76.12) |  |  |
|  | <42.41 | 4(5.97) | 63(94.03) |  |  |

Abbreviation: VT, tidal volume; MV, minute ventilation; ERV, expiratory reserve volume; FVC, forced vital capacity; FEV1, forced expiratory volume in 1 s; PEF, Peak expiratory flow; MEF75%, maximal expiratory flow at 75% of FVC; MEF50%, maximal expiratory flow at 50% of FVC; MEF25%, maximal expiratory flow at 25% of FVC; MMEF, maximal mid-expiratory flow; MVV, maximal voluntary ventilation.


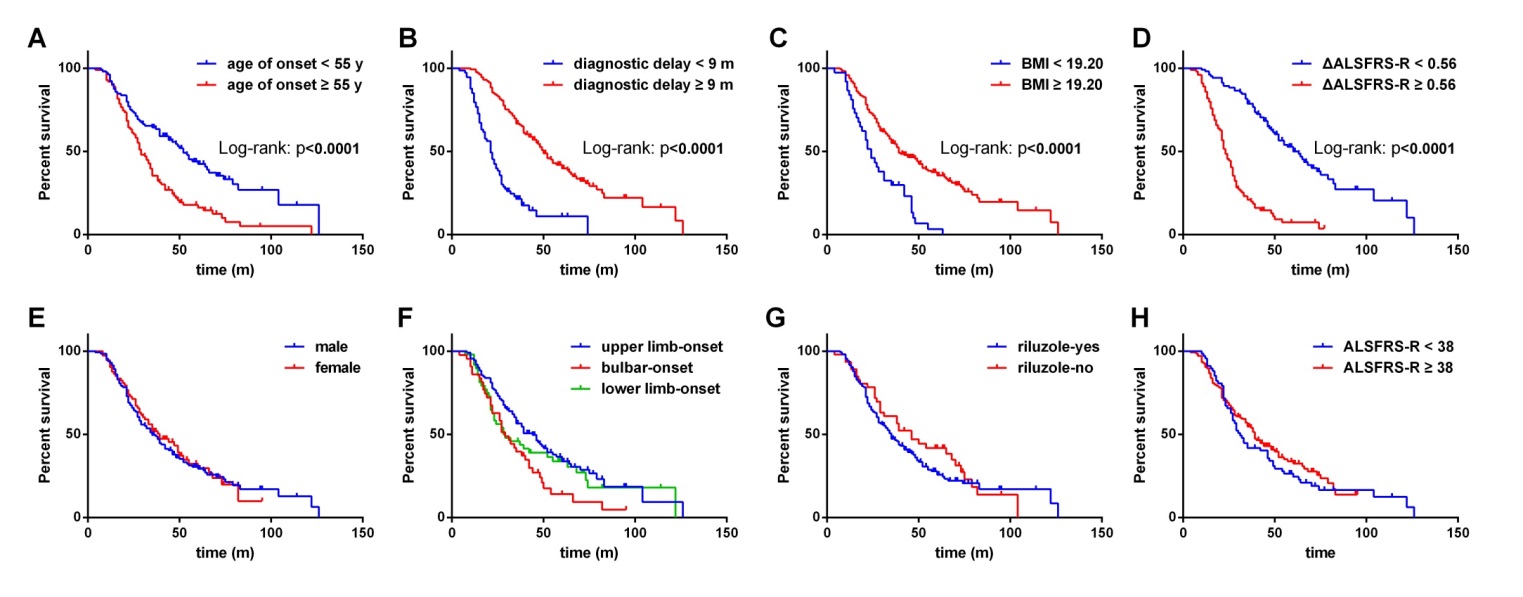


Figure S1 Kaplan-Meier survival curves of ALS patients with age of onset < 55 y vs. ≥ 55 y (A), diagnostic delay < 9 m vs. ≥ 9 m (B), BMI < 19.20 vs. ≥ 19.20 (C), ΔALSFRS-R < 0.56 vs. ≥ 0.56 (D), gender (E), site of onset (F), use of riluzole (G), and ALSFRS-R score < 38 vs. ≥38 (H).


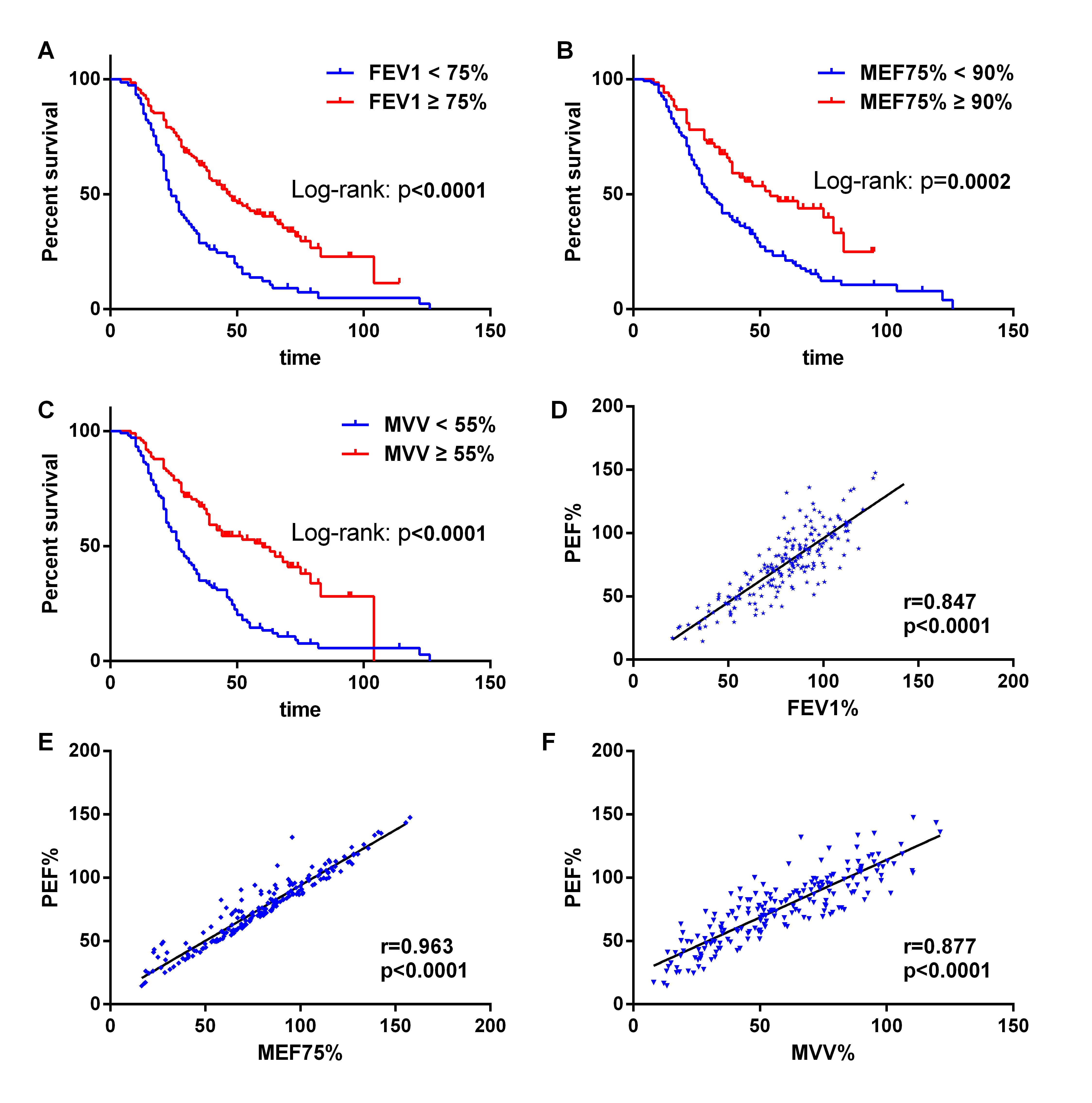


Figure S2. Kaplan-Meier survival curves of ALS patients with FEV1 < 75% vs. FEV1 ≥ 75% (A), MEF75% < 90% vs. MEF75% ≥ 90% (B), and MVV < 55% vs. MVV ≥ 55% (C). Correlation analysis of PEF and FEV1 (D), PEF and MEF75% (E), PEF and MVV (F).


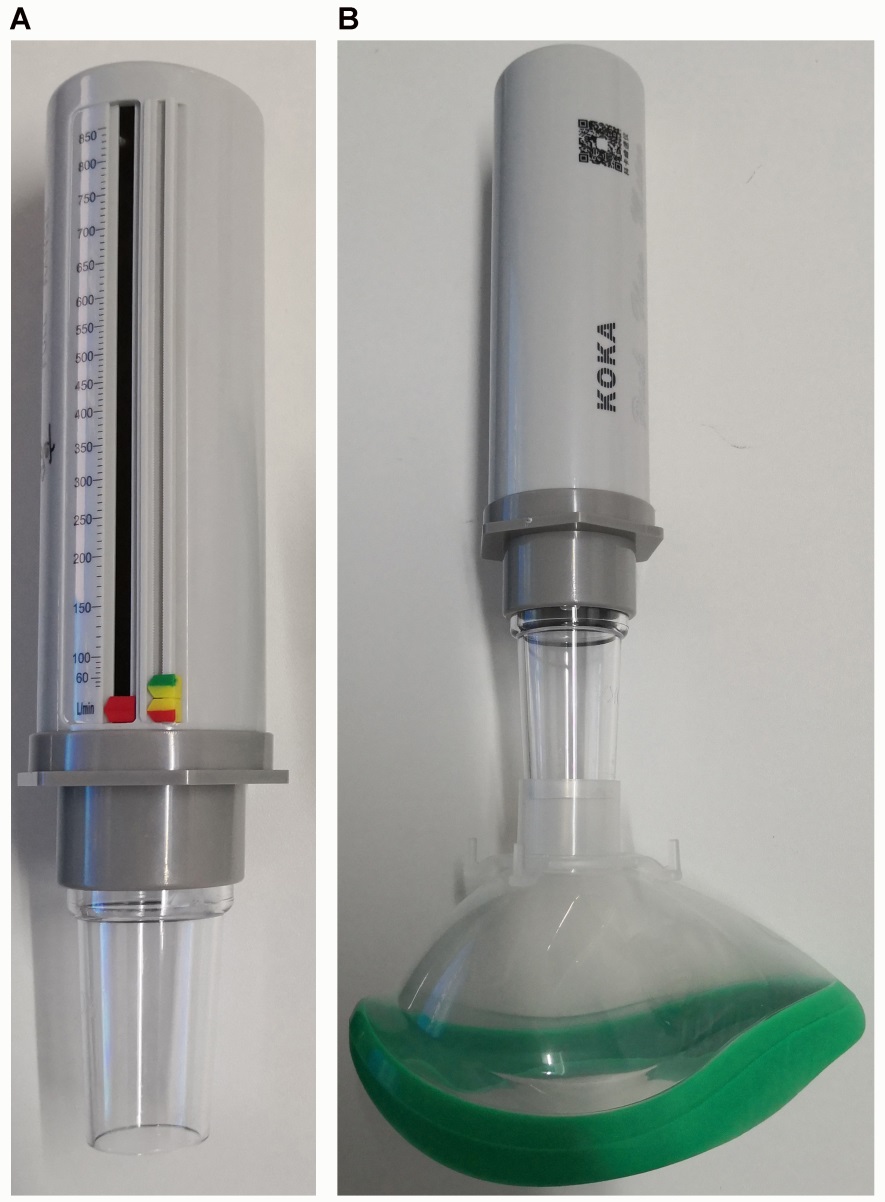


Figure S3 (A) The household peak flow meter (KOKA). (B) The revised peak flow meter with face mask.
